# Outcomes following the implementation of a quality control campaign to decrease sternal wound infections after coronary artery by-pass grafting

**DOI:** 10.1186/s12872-015-0148-4

**Published:** 2015-11-17

**Authors:** Rickard P. F. Lindblom, Birgitta Lytsy, Camilla Sandström, Nadjira Ligata, Beata Larsson, Ulrika Ransjö, Christine Leo Swenne

**Affiliations:** Department of Cardiothoracic Surgery and Anesthesia, Uppsala University Hospital, 751 85 Uppsala, Sweden; Department of Medical Sciences, Unit for Clinical Microbiology and Infectious Medicine, Uppsala University, Uppsala, Sweden; Department of Public Health and Caring Sciences, Uppsala University, Uppsala, Sweden

**Keywords:** CABG, Sternal wound infection, Quality control campaign

## Abstract

**Background:**

Coronary artery by-pass grafting (CABG) remains the optimal strategy in achieving complete revascularization in patients with complex coronary artery disease. However, sternal wound infections (SWI), especially deep SWI are potentially severe complications to the surgery. At the department of cardiothoracic surgery in Uppsala University Hospital a gradual increase in all types of SWI occurred, which peaked in 2009. This prompted an in-depth revision of the whole surgical process. To monitor the frequency of post-operative infections all patients receive a questionnaire that enquires whether any treatment for wound infection has been carried out.

**Methods:**

All patients operated with isolated CABG between start of 2006 and end of 2012 were included in the study. 1515 of 1642 patients answered and returned the questionnaire (92.3 %). The study period is divided into the time before the intervention program was implemented (2006-early 2010) and the time after the intervention (early 2010- end 2012). To assess whether potential differences in frequency of SWI were a consequence of change in the characteristics of the patient population rather than an effect of the intervention a retrospective assessment of medical records was performed, where multiple of the most known risk factors for developing SWI were studied.

**Results:**

We noticed a clear decrease in the frequency of SWI after the intervention. This was not a consequence of a healthier population.

**Conclusions:**

Our results from implementing the intervention program are positive in that they reduce the number of SWI. As several changes in the perioperative care were introduced simultaneously we cannot deduce which is the most effective.

**Electronic supplementary material:**

The online version of this article (doi:10.1186/s12872-015-0148-4) contains supplementary material, which is available to authorized users.

## Background

Complete revascularization in patients with severe coronary artery disease is optimally achieved with coronary artery bypass-grafting (CABG) [1–3]. However, CABG is also vitiated with complications, out of which one of the most serious is sternal wound infection (SWI) [4]. SWI leads to both increased patient morbidity and mortality as well as augmented health care costs. Compared to a CABG operation without SWI the median cost of a single CABG procedure with SWI was $49.449 compared to $18.218 [5]. The incidence of reported SWI following CABG is highly variable due to differences in patient profiles and surgical techniques. There are also differences in classification of the SWI, where most studies have focused on the more severe, deep infections which typically occur in 1–2 % of CABG operations [4] others also report more superficial infections which are more common, with incidences between 5–12.0 % [6, 7].

There are a number of known patient-related, pre- intra- and postoperative risk factors associated with increased risk of developing SWI after CABG which have been studied extensively [6, 8]. Accordingly, major attempts have been made to decrease the incidence of SWI. It is reasonable to believe that strict adherence to good hygienic routines in the perioperative period is beneficial, and there is documented evidence of the efficacy of hygiene interventions in the setting of cardiac surgery [9, 10]. However, these studies were small (less than 200 patients), spanned short periods of time (1–2 years) and included mixed patient groups, i.e. different kinds of open heart procedures. Also, there are multiple challenges to overcome when introducing changes in organizations [11]. In particular the leadership is challenged and methods of evaluating the performance of the clinic are of essence. Health care processes are complex and multivariate. It is therefore recommended to analyze the events when a patient is harmed or severely threatened to be so within the health care process using a standardized method, for instance a root cause analysis (The Swedish National board of Health and Welfare, http://www.socialstyrelsen.se/patientsakerhet/ledningssystem/analyserarisker). The focus of continuous event analysis should be on system aspects and organizational issues and complications identified related to the surgical care should be recorded continuously [11]. Furthermore, collaboration with support functions and other professions constitutes an important step in improving medical organizations [12].

During 2009 especially the number of deep, but also superficial, postoperative sternal wound infections within 30 days of surgery increased dramatically at the Department of Cardiothoracic Surgery and Anesthesia, Uppsala University Hospital. This was considered a serious problem and an extensive event analysis was performed. The aim of the current study was to assess how the semi-structured quality interventions that were implemented following the campaign impacted on the number of self-reported SWI 30 days after CABG.

## Methods

### Data collection

The study was performed at the department of Cardiothoracic Surgery and Anesthesia, Uppsala University Hospital, Sweden. The unit performs around 700 open heart surgeries annually out of which 250 to 300 are isolated CABG. During the period between the start 2006 and the end of 2012 1642 patients underwent isolated CABG. To evaluate the incidence of sternal wound infection 30 days after surgery a prospective, post-discharge patient registered evaluation was performed (Additional files 1 and 2). The infections were self-reported and the response rate was high throughout the study period; 1515 of 1642 patients (92.3 % response rate) answered the post-discharge evaluation. Any kind of postoperative SWI (superficial, subcutaneous, to the sternum or deep/mediastinitis) that warranted active treatment with either/or antibiotics or surgical revision was included. 1334 (81.2) of the 1642 patients were men and 308 women (18.8 %). The mean age was 67 ± 9 years.

**Fig. 1:**
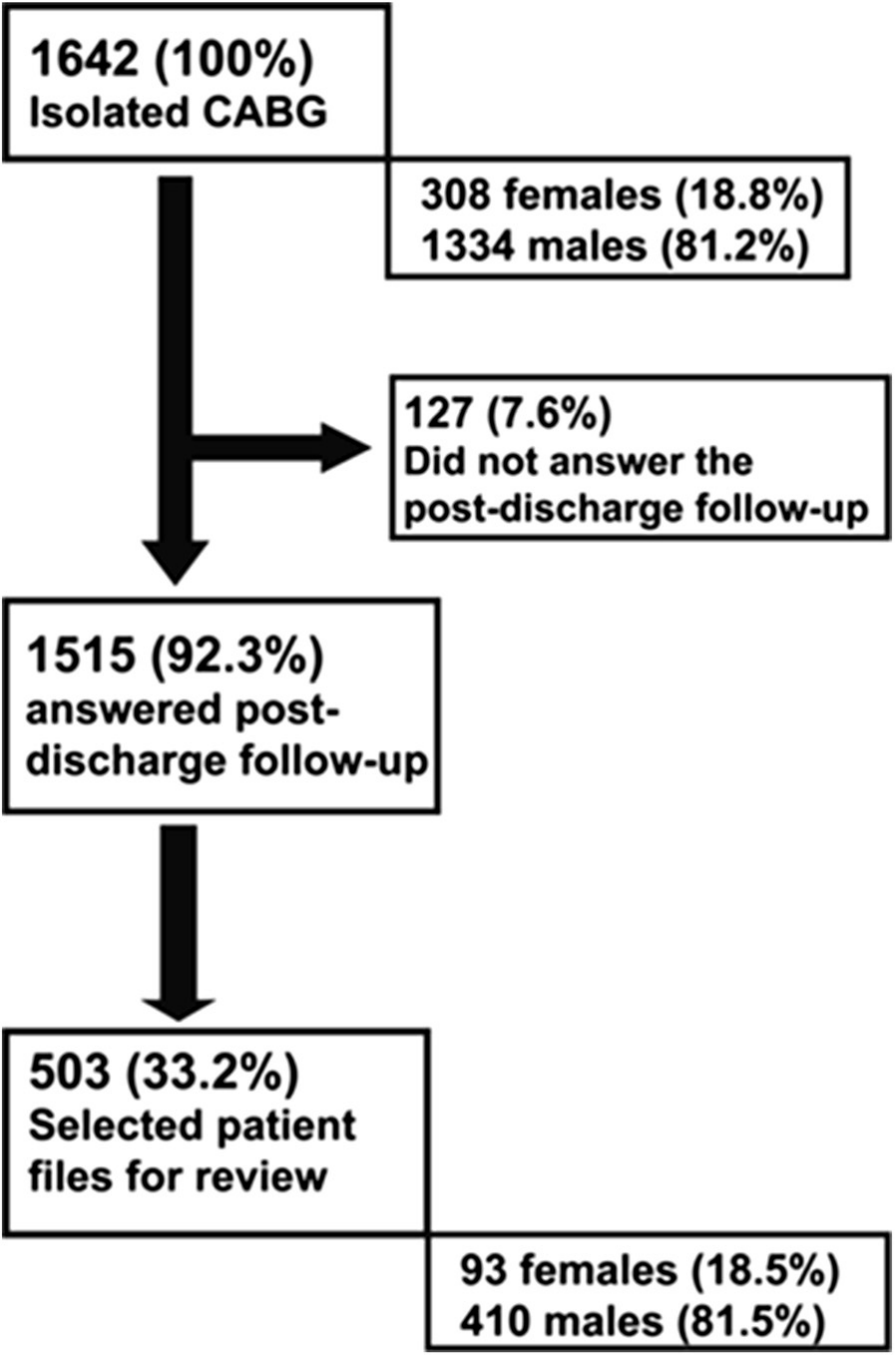
Illustration of the inclusion process

To perform a more detailed study of potential risk factors for SWI and better characterize the population a retrospective review of the medical records of every third patient, in chronological order of operation date, that had answered the post-discharge evaluation was selected and included in a separate cohort (Fig. 1). 503 of 1515 (33.2 %) patients were eligible for records review, the characteristics of this cohort were similar to the whole study population regarding age and sex (age 67 ± 9, M/F 410/93, 81.5/18.5 %). A chronological sampling, as opposed to stricter randomization, was chosen in order to reflect the seasonal fluctuations in operation volume and infection frequency [13].

The review of the 503 medical records included specific studies of patient-related risk factors such as age, gender, body mass index (kg/m^2^), preoperative insulin-like growth factor 1 (IGF-1) levels, diabetes, preoperative hemoglobin concentrations, preoperative serum creatinine concentrations, chronic obstructive pulmonary disease (COPD), corticoid steroid treatment, current smoking status and preoperative ejection fraction (EF). The review of intraoperative risk factors included the number of saphenous vein grafts and internal mammary arteries used and the duration of cardiopulmonary by-pass and aortic cross clamp time. The review of postoperative risk factors included if erythrocyte transfusions had been given on day 0, 1 and 2 and also assessment of mean blood glucose concentration day 0, 1 and 2. The day of operation was denoted as day 0 and day 1 started 6 am in the morning after surgery.

### Hygiene routines before intervention

#### Preoperatively

Elective and semi-urgent cases were normally admitted to the hospital the day before surgery, but sometimes earlier. The patients had one shower at home and one shower at the hospital. Hair removal on the leg and chest hair was done using a disposable clipping machine the night before surgery.

#### Intraoperatively

In the operating room, the skin was scrubbed with 0.5 % chlorhexidine in 70 % ethanol, which was allowed to air dry. The surgical procedures were performed in an operating room with ultra clean air with 10 cfu/m^3^. The staff wore tightly woven scrub suits with cuffs and helmets tucked under the neckline of the shirt. Cloxacillin, 2 g, was administered intravenously three times a day for 2 days, starting in the morning of surgery.

#### Postoperatively

During the study period the policy of normalization of blood glucose concentrations in the immediate postoperative period changed; for patients without diabetes it was 5–7 mmol/l in 2006 to 2007 and 4–6 mmol/l from 2007 and onwards. For patients with diabetes the goal was 5–7 mmol/l in 2008 to 2011. Wound dressings were in the first period (2006–2010) allowed to stay in place for 4 days if dry.

### Changes in hygiene routines after intervention

When the increase in SWI was detected an action plan was devised. Representatives from all personnel categories (surgeons, anaesthesiologists, nurses and nurses aids) from all three units (operation, intensive care unit and ward unit) of the department as well as from the departments of Clinical Microbiology and Infectious Medicine met at regular intervals during September 2009–April 2010 to perform a structured root cause analysis for infection according to The National board of Health and Welfare (http://www.socialstyrelsen.se/patientsakerhet/ledningssystem/analyserarisker) and [14]*.* All elective operations ceased for 1 week. The changes in routines are summarized below.

#### Preoperatively

The preoperative washing routines were changed to two showers and scrubbing with a 4 % chlorhexidine detergent solution at the hospital. In the elective cases, care was taken to admit the patients to the ward only on the day before surgery, with a polyclinical meeting with a surgeon several days before admittance in order to spend as little time possible preoperatively in the hospital.

#### Intraoperatively

After the intervention the routine for antibiotic prophylaxis changed; Cloxacillin 2 g was instead administered four to five times all in the day of surgery, every four hours. The first dose started 30–60 min before the skin incision to achieve the maximum concentration in the blood at time of surgery.

A general policy of increased overall discipline in the operating theatre, which aimed at restricting the number of door-openings and minimizing the number of people in the operating theatre (maximum 11) was enforced. Regarding surgical technique, a standardized closure of the sternotomy with preferably eight single sternal wires that were not in figure-of-eights was introduced for all surgeons. There are no RCT’s comparing closure techniques, although there is some evidence from biomechanical studies that supports the use of single wires as compared to the figure-of-eight configuration [15, 16]. All surgeons were urged to wear double gloves. Also, the WHO checklist for Safe surgery was introduced [17]. At the end of surgery the skin around the incision was scrubbed with 0.5 % chlorhexidine in 70 % ethanol before applying the wound dressing. Lastly, a revision of cleaning procedures for the operation theatre and of reusable materials was done with purchase of disinfectable keyboards and more disposable materials.

#### Postoperatively

In the second period (2010–2012) all sterile wound dressings were removed after 48 h and the wound inspected. No new dressing was added after this unless the wound was still open in which case a new dressing was placed after washing with sterile saline, the wound was then inspected the next day again and the procedure repeated if necessary. For patients with diabetes the blood glucose goal was changed to 6–8 mmol/l from 2011 and onwards

#### General attitudes

In general, a higher level of alertness and adherence to general and basic hand hygiene routines was enforced after the intervention as well as the introduction of disposable plastic aprons to be worn during patient contact. To ensure compliance general unannounced inspections were performed throughout the clinic at regular intervals and feed-back given to the staff, in all categories.

### Statistics

Possible predictors for SWI were compared in patients with and without SWI. Nominal data were analysed using the *X*2 test. The Kolmogorov–Smirnov goodness-of-fit test was used to analyse data distribution for the remaining risk factors. If distributions deviated significantly (*p* < 0.05) from the normal distribution the data were analysed using the Mann–Whitney U or Kruskal–Wallis tests. Those variables not deviating from the normal distributions were analysed using Student’s *t*-test or one-way analysis of variance (ANOVA). Variables are given as mean (and SD, standard deviation), categorical variables as percentages.

### Ethics, consent and permissions

The current work is the result of quality improvement work performed at Department of Cardiothoracic Surgery and Anesthesia, Uppsala University Hospital, Sweden and adheres to the Declaration of Helsinki [18] as well as national and local ethical guidelines for research http://www.codex.vr.se. Written, informed content was obtained from all participants.

## Results

### Patient demographics

Initial analysis aimed to exclude any gross differences in composition between the patient cohort selected for detailed studies of medical records (*n* = 503), and the whole study population during the period, i.e. the 1642 patients operated with CABG between 2006 and end of 2012. The populations had almost identical mean age and the distribution between sexes was similar (Table 1 and Fig. 1).

**Table 1:** Comparison between the population selected for detailed review with the whole study population (*n* = 1642)

	2006–2012		
	Review of patient medical records (*n* = 503)	No review of patient medical records (*n* = 1139)	*p*
Age (years)	67 ± 9	67 ± 9	0.907
Sex M/F	410/93	923/241	0.874

### Identification of risk factors associated with sternal wound infection in the cohort

The characteristics of the cohort of patients that were selected for retrospective analysis of medical records are described in Table 2, where the patients that suffered SWI are compared to the patients that did not suffer SWI, over the whole study period. This revealed that the patients that suffered from SWI more often had diabetes (*p* = 0.022) and more often received blood transfusions on postoperative day 2 (*p* = 0.007) (Table 2). There was a strong trend (*p* = 0.086) that lower preoperative hemoglobin and higher levels of preoperative IGF-1 (*p* = 0.070) were more prevalent in the SWI group. Noteworthy is that the SWI group had a tendency to be younger than the non-infected group (*p* = 0.053) (Table 2).

**Table 2:** Characteristics of the infected and non-infected groups (*n* = 503) seen over the whole study period

	Patients without infection (*n* = 455)	Patients with infections (*n* = 48)	*p*-value
Patient related riskfactors			
Age (years)	67 ± 9	65 ± 10	*0.053*
Sex M/F	374/81	36/12	*0.222*
Body mass index (kg/m^2^)	28 ± 4	28 ± 4	*0.954*
Preoperative insulin-like growth factor 1 (mmol/mol)	45 ± 11 (*n* = 438)	51 ± 17 (*n* = 46)	*0.070*
Diabetes (%)	119/455 (26.5 %)	20/48 (41.7 %)	0.022 (*)
Preoperative hemoglobin concentrations (g/L)	141 ± 14 (451)	137 ± 11 (*n* = 47)	*0.086*
Preoperative serum creatinine concentrations (μg/L)	86 ± 28 (*n* = 452)	85 ± 23 (*n* = 47)	*0.597*
Corticoid steroid treatment (%)	12/455 (2.6 %)	2/48 (4.2 %)	*0.540*
Current smoking no/smoked the last 2 months/never smoked	54/314/87	7/36/5	*0.319*
Chronic Obstructive Pulmonary Disease (%)	12/455 (2.6 %)	1/48 (2.1 %)	*0.818*
Ejection fraction (%)	48 ± 9 (*n* = 348)	49 ± 10 (*n* = 35)	*0.272*
Intraoperative risk factors			
Number of saphenous vein grafts (n)	3 ± 1 (*n* = 424)	3 ± 1	*0.693*
Internal mammary artery 0/1/2	41/412/2	2/46/0	*0.621*
Duration of cardiopulmonary by-pass time (min)	85 ± 29 (*n* = 453)	85 ± 27	*0.683*
Duration of aortic cross clamp time (min)	47 ± 19 (*n* = 453)	49 ± 19	*0.634*
Postoperative risk factors			
Erythrocyte transfusion day 0 (%)	135/455 (29.7 %)	19/48 (39.6 %)	*0.156*
Erythrocyte transfusion day 1 (%)	71/455 (15.6 %)	10/48 (20.1 %)	*0.349*
Erythrocyte transfusion day 2 (%)	65/455 (14.3 %)	14/48 (29.2 %)	0.007 (**)
Mean blood glucose concentration day 0 (mmol/L)	7.8 ± 1.3 (*n* = 453)	7.5 ± 1.0	*0.295*
Mean blood glucose concentration day 1 (mmol/L)	8.2 ± 1.6 (*n* = 453)	8.5 ± 1.8	*0.213*
Mean blood glucose concentration day 2 (mmol/L)	9.0 ± 2.2 (*n* = 380)	9.4 ± 3.0 (*n* = 46)	*0.788*

*= p <0.05, **= p <0.01, **=p <0.001

### Patient profiles in period one compared to period two

To exclude that any potential differences in the rates of SWI following CABG in the two study periods, i.e. before and after the intervention, were an epiphenomena due to differences in the study populations, assessment of the profiles of the patient populations in the two periods was performed. We therefore compared the whole groups, i.e. both infected and non-infected patients grouped together, from the two periods. This suggested that the two study populations were largely similar with regard to patient-related factors (Table 3). But there were some differences, for instance in period one the preoperative IGF-1 was slightly, but significantly higher, on the other hand, there were significantly more patients in period two that were on corticosteroid treatment. The groups were also similar with regard to intraoperative factors (Table 3). However, in the postoperative parameters there was one major difference between the groups, and this was the amount of blood transfusions had significantly been reduced, from 18 to 9 %. Another minor difference was that the perioperative blood glucose was slightly higher in the second period (Table 3).

**Table 3:** Risk factors for self-reported sternal wound infection after coronary artery by-pass graft surgery in patients with review of medical records (*n* = 503), comparison between into period one and two

	2006–2010 to week 26 (Period 1)	2010 from week 27–2012 (Period 2)	*p*-value
	*n* = 362	*n* = 141	
Patient related riskfactors			
Age (years)	67 ± 9	67 ± 9	*0.610*
Sex M/F	73/289	121/20	*0.121*
Body mass index (kg/m^2^)	28 ± 4	28 ± 4	*0.998*
Preoperative insulin-like growth factor 1 (mmol/mol)	46 ± 12	44 ± 11	0.014 (*)
Diabetes (%)	94/268 (26 %)	45/96 (32 %)	*0.180*
Preoperative hemoglobin concentrations (g/L)	140 ± 14	141 ± 14	*0.880*
Preoperative serum creatinine concentrations (μg/L)	86 ± 28	87 ± 38	*0.747*
Chronic obstructive pulmonary disease (%)	12/350 (3.3 %)	1/140 (0.7 %)	*0.098*
Corticoid steroid treatment (%)	6/356 (1.7 %)	8/133 (5.7 %)	0.014 (*)
Current smoking/smoked the last 2 months/never smoked	46/246/70	15/104/22	*0.444*
Ejection fraction (%)	48 ± 9	49 ± 8	0.041 (*)
Intraoperative risk factors			
Number of saphenous vein grafts (n)	3 ± 1	3 ± 1	*0.982*
Internal mammary artery 0/1/2	30/330/2	13/128/0	*0.646*
Duration of cardiopulmonary by-pass time (min)	85 ± 28	85 ± 29	*0.744*
Duration of aortic cross clamp time (min)	47 ± 19	48 ± 20	*0.818*
Postoperative risk factors			
Erythrocyte transfusion day 0 (%)	118/244 (32.6 %)	36/105 (25.5 %)	*0.123*
Erythrocyte transfusion day 1 (%)	61/301 (16.9 %)	20/121 (14.2 %)	*0.465*
Erythrocyte transfusion day 2 (%)	66/296 (18.2 %)	13/128 (9.2 %)	0.013 (*)
Mean blood glucose concentration day 0 (mmol/L)	7.7 ± 1.3	8.2 ± 1.7	0.000 (***)
Mean blood glucose concentration day 1 (mmol/L)	8.2 ± 1.6	8.3 ± 1.8	*0.408*
Mean blood glucose concentration day 2 (mmol/L)	9.0 ± 2.3	8.9 ± 2.3	*0.634*

*= p <0.05, **= p <0.01, **=p <0.001

### Effect of the hygienic intervention of the frequency of SWI

We next assessed whether the intervention program decreased the number of postoperative SWI; this was done by comparing the incidence of SWI in period one, before the intervention, with period two, after the intervention. This demonstrated that the frequency of all kinds of SWI, that is both deep and superficial, decreased from 12.2 % in period one to 2.8 % (Table 4) in period two.

**Table 4:** Risk factors for self-reported sternal wound infection after coronary artery by-pass graft surgery in patients with review of medical records (*n* = 503), comparison between into period one and two divided into infected and non-infected patients

	2006–2010 to week 26 *n* = 362			2010 from week 27–2012 *n* = 141		
	Patients without infection (*n* = 318) 87.8 %	Patients with infections (*n* = 44) 12.2 %	*p*-value	Patients without infection (*n* = 137) 97.2 %	Patients with infections (*n* = 4) 2.8 %	*p*-value
Patient related riskfactors						
Age (years)	67 ± 9	65 ± 10	*0.198*	68 ± 9	56 ± 7	0.032 (*)
Sex M/F	256/62	33/11	*0.394*	118/19	3/1	*0.529*
Body mass index (kg/m^2^)	28 ± 4	27.1 ± 4	*0.606*	28 ± 4	32 ± 4	*0.051*
Preoperative insulin-like growth factor 1 (mmol/mol)	46 ± 11 (*n* = 307)	52 ± 18 (*n* = 42)	*0.115*	44 ± 11 (*n* = 131)	44 ± 8	*0.917*
Diabetes (%)	75/318 (23.6 %)	19/44 43.2 %	0.005 (*)	44/137 (32.1 %)	1/4 (25 %)	*0.763*
Preoperative hemoglobin concentrations (g/L)	141 ± 14 (*n* = 315)	137 ± 11 (*n* = 43)	*0.115*	141 ± 14 (*n* = 136)	136 ± 11	*0.467*
Preoperative serum creatinine concentrations (μg/L)	86 ± 22 (*n* = 243)	86 ± 23 (*n* = 43)	*0.908*	87 ± 39	70 ± 11	*0.094*
Chronic obstructive pulmonary disease (%)	11/318 (3.5 %)	1/44 (2.3 %)	*0.680*	1/137 (0.7 %)	0/4	*0.864*
Corticoid steroid treatment (%)	5/318 (1.6 %)	1/44 (2.3 %)	*0.733*	7/137 (5.1 %)	1/4 (25 %)	*0.090*
Current smoking/smoked the last 2 months/never smoked	39/211 /68	7/35/2	0.029 (*)	15/103/19	0/1/3	0.004 (**)
Ejection fraction (%)	47 ± 10 (*n* = 217)	49 ± 11 (*n* = 31)	*0.222*	49 ± 8 (*n* = 131)	51 ± 7	*0.481*
Intra-operative risk factors						
Number of saphenous vein grafts (n)	3 ± 1 (*n* = 317)	3 ± 1	*0.893*	3 ± 1 (107)	4 ± 1	*0.117*
Internal mammary artery 0/1/2	28/288/2	2/42/0	*0.675*	13/124/0	0/4/0	*0.518*
Duration of cardiopulmonary by-pass time (min)	84 ± 2 (*n* = 317)	84 ± 28	*0.886*	85 ± 29 (*n* = 136)	90 ± 6	*0.748*
Duration of aortic cross clamp time (min)	47 ± 18 (*n* = 317)	48 ± 19	*0.929*	48 ± 20 (*n* = 136)	53 ± 6	*0.582*
Postoperative risk factors						
Erythrocyte transfusion day 0 (%)	100/318 (31.4 %)	18/44 (40.9 %)	*0.209*	35/137 (25.5 %)	1/4 (25 %)	*0.980*
Erythrocyte transfusion day 1 (%)	52/318 (16.4 %)	9/44 (20.5 %)	*0.496*	19/137 (13.9 %)	1/4 (25 %)	*0.529*
Erythrocyte transfusion day 2 (%)	53/318 (16.7 %)	13/44 (29.5 %)	0.038 (*)	12/137 (8.8 %)	1/4 (25 %)	*0.268*
Mean blood glucose concentration day 0 (mmol/L)	7.5 ± 1.1 (*n* = 317)	7.5 ± 1.0	*0.916*	8.2 ± 1.7 (*n* = 136)	7.6 ± 0.4	*0.585*
Mean blood glucose concentration day 1 (mmol/L)	8.1 ± 1.4 (*n* = 316)	8.5 ± 1.9	*0.504*	8.3 ± 1.8	8.1 ± 0.9	*0.821*
Mean blood glucose concentration day 2 (mmol/L)	9.0 ± 2.1 (*n* = 267)	9.4 ± 3.1 (*n* = 42)	*0.902*	8.9 ± 2.4 (*n* = 113)	9.1 ± 1.9	*0.887*

*= p <0.05, **= p <0.01, **=p <0.001

In period two the patients that suffered from SWI were significantly younger than the healthy group, whereas in period one there was no age difference between the infected and non-infected (Table 4). Smoking was a risk factor for developing SWI throughout the study period (Table 4). Viewed over the whole study period, diabetes was more common in the infected patients (Table 2), whereas after the intervention there was no significant difference in the frequency of SWI between the patients with or without diabetes (Table 4), although the number of infected patients was small. In period one erythrocyte transfusion on day 2 was associated with increased risk of SWI, whereas this was not significant in period two (Table 4).

### Microbial flora over time

Lastly, we investigated whether the hygienic intervention could cause a shift or selection in the pathogenic microflora involved in the SWI. The results are more thoroughly described in (Lytsy et al. [19], in press doi:10.1016/j.jhin.2015.08.021). In summary there was no difference in the proportion of the two most common pathogens *Staphylococcus aureus* and Coagulase Negative Staphylococci in the pre- and post-intervention wound cultures.

## Discussion

In this study we demonstrate our experience from implementing an intervention program on reducing the incidence of sternal wound infection following CABG. A methodological and structured root cause analysis was performed according to a standardized protocol. This meant that a cross-professional review of the medical-, care- and hygiene procedures, with a system of continuous monitoring and feed-back was introduced. The discussion and work that followed are continuous processes that ultimately aim to enforce a higher level of alertness’ to all staff in the optimization of the care of the patient. The aim of the current study was not to primarily study risk factors for developing SWI after CABG, as this has been extensively performed before, but to assess whether the intervention was meaningful, and to exclude that potential differences were instead caused by a change in patient demographics.

### Risk factor profiling

Analysis of the general risk factor profile of the patients demonstrated that the burden of co-morbidities generally associated with increased risk of postoperative infection were not markedly different in period one compared to period two, except for that there were half as many blood transfusions in period two as in period one. This probably contributes to some of the improvement seen in the frequency of SWI after the hygienic intervention. Re-operation for bleeding is a known risk-factor for developing SWI [19], and the reduction in the number of blood transfusions could potentially have reflected a reduced number of re-explorations for bleeding. However, when reviewing the medical records of the 48 patients that suffered SWI, none of them were re-operated for bleeding. So, the reduction in blood transfusion does not reflect a reduced re-operation for bleeding in the infected group. But blood transfusion *per se* has also been linked to development of mediastinitis, often synonymous with DSWI [21]. Consequently, the decline in transfusion rates in period two may have contributed to the decrease in postoperative infections, however, the 50 % reduction in blood transfusions does not solely explain the almost 80 % reduction in SWI. This suggests that the action program had significant additional effect, on top of that which could be attributed reduced transfusion. It also suggests that introduction of the action program as a positive side-effect reduced the amount of blood transfusions, which in any case is a desirable consequence.

Furthermore we saw that diabetes and erythrocyte transfusions on day 2 after surgery were no longer associated with increased risk of SWI after the intervention, suggesting that the changes implemented perhaps were especially effective for these patients, although these numbers should be interpreted with caution as the infected group in period two is small.

We did not see any association between higher blood glucose and SWI, although the general ambition of controlling blood glucose levels is high at our department and both the infected and non-infected groups were well regulated in this regard. With this said, it is therefore still possible that poor glycemic control may be a risk for SWI [22], although contradicting evidence exists, as intense perioperative glucose monitoring has been shown to not affect the rates of wound infection following cardiac surgery, but to decrease mortality [23]. Other commonly studied factors like cross-clamp time or ECC time were in our data not significant risk factors for developing SWI.

The tendency for lower age in the infected group as compared to the healthy group is somewhat surprising, but could reflect a dimension not mirrored by the other parameters, for instance genetic factors [24]. And since they are operated with CABG at a young age, this may suggest a more aggressive disease, rather than young age being a risk factor *per se* for suffering SWI.

In summary, the selected risk factors that proved to differ significantly between the infected and healthy group were not new, although IGF-1 seems an interesting molecule to study further in these contexts, as there was a strong trend (*p* = 0.07) for higher levels in the infected group. There is to our knowledge no studies assessing the link between high levels of IGF-1 and SWI. IGF-1 is a good marker for nutritional status and anabolic responses [25] and IGF-1 is often considered beneficial in wound healing [26, 27], even though it has been shown detrimental in parasitic skin infections [28]. IGF-1 is also strongly linked to glucose-insulin pathways, however, the link to diabetes is not altogether easy, as both high and low levels of IGF-1 have been shown to correlate with subsequent onset of diabetes [29]. Also, alterations in the IGF-1 gene leads to lower levels of IGF-1, in turn linked to a higher risk of developing both diabetes and myocardial infarction [30]. The strong trend for elevated levels of IGF-1 in the SWI group are therefore difficult to interpret. And although we believe that, in our setting, elevated levels of IGF-1 could be seen as a marker for poor metabolic control, this may be an oversimplification, and instead reflect something more complex.

A general impression at the clinic was that the proportion of *S.aureus* decreased and the proportion of CoNS increased in the infected wounds after the intervention. However, statistical analysis of the wound cultures from the patients that were surgically revised revealed no differences, as further discussed in (Lytsy et al. 2015, in press doi:10.1016/j.jhin.2015.08.021). However, we lack wound cultures from a majority of the superficial SWI, as they were treated at other hospitals than Uppsala University Hospital, and we can therefore not say if the intervention affected the microbial population in the superficial SWI. This constitutes an interesting question for further studies, especially as it has been shown that the use of a 4 % chlorhexidine detergent solution can reduce the amount of *S.aureus* colonization [31]. Even though this was in the setting of pediatrics it could suggest that the extra preoperative shower with chlorhexidine that was added as a part of the intervention could lead to less *S.aureus* infections. Regarding chlorhexidine skin scrub, there is also evidence from orthopedic surgery that postoperative scrub of the wound with chlorhexidine may be effective in reducing wound infections [32]. So we believe the chlorhexidine skin scrub at the end of surgery was an easy and cost-effective measure to introduce, which may have contributed to the beneficial outcome.

### The complexity of assessing interventions

A weakness with the study is that the findings are correlative, as no randomization or control group existed. Even though the importance of hygiene in the setting of surgery cannot be considered controversial, the degree of impact can be discussed. The present study therefore to some grade objectifies the significance of hygienic interventions. Another aspect is that we have not analyzed the cost-effectiveness of the program, merely the medical aspects. Although we deem it most likely that the undertaken actions have been a good investment, not only in decreased patient suffering, but also economically as the cost of a CABG with postoperative deep SWI is almost 3-fold to that of a CABG without SWI [5], and many of the introduced changes did not cost extra. However, this could be the topic of further research.

It is difficult to assess the influence of individual interventions when multiple changes are introduced simultaneously. We can therefore not say which of the changes in the perioperative routines had the most significant effect. It is possible that some of the changes *de facto* were negative, but that this penetrance could have been blunted by the stronger positive effect of another change. It is therefore important to constantly aim to improve, and not settle for a final solution. To ultimately study the impact of individual actions randomized clinical trials are warranted.

As often with patient-based collection of data there is a risk for selection bias; it is possible that the patients that suffered from SWI in a higher extent chose, or were not able, to respond to the post-discharge evaluation. But for the patients that were re-admitted for surgery, i.e. the most severe infections, this data exists and is reliable, no matter if the patient answered the evaluation or not. Also, the response rate was very good throughout the study period, with no clear yearly variation. We therefore find it reasonable to believe that the frequency of non-responders were the same in the two periods, and that the potential selection bias would affect both periods similarly.

It should also be noted that the study includes all kinds of SWI which means that a large spectrum of disease severity exists within the infected group. However, main the purpose of the current study was not to specifically study SWI morbidity or mortality, which has been well studied before, but rather to evaluate the effect of the intervention on the frequency of all types, i.e. both superficial and deep, postoperative SWI. Ultimately all kinds of SWI are best avoided, both for the sake of the patients and the health care system.

## Conclusions

The risk factors associated with developing SWI after CABG are well studied but still not fully understood, and tend to differ between materials [8]. The aim of the current study was not to primarily study risk factors, this was done with the goal to exclude that any differences in outcome/SWI after the intervention were confounded by differences in the study populations. This was to some extent the case as the number of blood transfusions were significantly reduced after the intervention. However, this did not explain the full difference, so it is still possible to conclude that the program had effect in reducing SWI, and as a positive side-effect reduced the number of blood transfusions.

The problem of SWI is multifactorial, and it is not possible to say where in the chain of events that surround a CABG operation the infectious course starts. We therefore believe it is of essence that all levels of the clinic, from operating surgeon to nurse assistant are engaged in the process. Another important aspect, and difficulty, is to maintain the increased discipline and accuracy with time, and not settle for a final solution, after the changes have been implemented and initial success achieved. With the current study we show that a structured root cause analysis and a purposeful and stringent intervention program can reduce the problem, although the exact importance of each single change is difficult to define.

## Additional files

Additional file 1:
**Questionnaire in Swedish, original.** (PDF 136 kb) Additional file 2:
**Questionnaire in English, translated.** (DOCX 13 kb)

## Abbreviations

CABG: Coronary artery by-pass grafting
SWI: Sternal wound infection
