# Supplementary material for: Outcomes following the implementation of a quality control campaign to decrease sternal wound infections after coronary artery by-pass grafting
Source: BMC Cardiovasc Disord. 2015 Nov 17;15:154. doi: 10.1186/s12872-015-0148-4 (PMC4650278; doi:10.1186/s12872-015-0148-4)
Supplement: Additional file 1: — Questionnaire in Swedish, original. (PDF 136 kb) [file 12872_2015_148_MOESM1_ESM.pdf]

AKADEMISKA SJUKHUSET

Personnummer ___________-________

Infektionsregistrering efter thoraxkirurgi vid Thoraxkirurgen, Akademiska sjukhuset i Uppsala

På Thoraxkirurgen bedrivs en fortlöpande uppföljning angående eventuella sårinfektioner efter kirurgi som en del i vårt kvalitetsarbete. Såret kan normalt ge ifrån sig en klar vätska som upphör efter en tid. Vid en infektion fortsätter det att rinna vätska från såret. Sårvätskan kan också ändra utseende och bli gulaktig i färgen och ibland lukta illa. Definitionen på sårinfektion är var man kan se för ögat.

Resultatet av uppföljningen ligger sedan till grund för översyn av våra rutiner i samband med operation. Genom att besvara enkäten medverkar Du till att vi skall kunna bedriva en så bra vård som möjligt. Dina uppgifter kommer att behandlas konfidentiellt och dina svar kommer inte att kunna identifieras i vår rapportering av sårinfektioner. Uppföljningen följer de bestämmelser som är föreskrivna i Personuppgiftslagen.

Frågor att besvara för dig som är hjärtopererad

Har Du under vårdtiden/efter utskrivningen från Thoraxkirurgen haft en infektion i såret på bröstkorgen?

ja nej

Har Du under vårdtiden/efter utskrivningen från Thoraxkirurgen haft en infektion där dränagen suttit?

ja nej

Nedanstående fråga besvaras av patienter som är opererade med Kranskärlskirurgi där man tagit kärl från benet

Har Du under vårdtiden/efter utskrivningen från Thoraxkirurgen haft en infektion i såret på benet?

ja nej

Nedanstående fråga besvaras av patienter där man tagit kärl från armen

Har Du under vårdtiden/efter utskrivningen från Thoraxkirurgen haft en infektion i såret på armen?

ja nej

Nedanstående fråga besvaras av patienter som är opererade i ljumsken

Har Du under vårdtiden/efter utskrivningen från Thoraxkirurgen haft en infektion i såret i ljumsken?

ja nej

Frågor att besvara för Dig som är lungopererad

Har Du under vårdtiden/efter utskrivningen från Thoraxkirurgen haft en infektion i operationssåret?

ja nej

Har Du under vårdtiden/efter utskrivningen från Thoraxkirurgen haft en infektion där dränagen suttit?

ja nej

Om Du har haft en infektion och svarat ja på någon av ovanstående frågor vill vi veta vilken vård Du fått.

Blev Du inlagd på sjukhuset på grund av Din infektion?

ja Vilket sjukhus? ______________________________ nej

Har man tagit en sårodling?

ja nej vet ej

Behandlades Du med antibiotika?

ja Namnet på antibotikan? _______________________ nej

Datum __________ ***Namn*** ________________________________

Om Du har frågor kontakta Christine Leo Swenne, Vårdutvecklare Onkologi-, thorax- och medicin divisionen, Tel. 018/611 39 94, e-post christine.leo.swenne@akademiska
