# Supplementary material for: Outcomes following the implementation of a quality control campaign to decrease sternal wound infections after coronary artery by-pass grafting
Source: BMC Cardiovasc Disord. 2015 Nov 17;15:154. doi: 10.1186/s12872-015-0148-4 (PMC4650278; doi:10.1186/s12872-015-0148-4)
Supplement: Additional file 2: — Questionnaire in English, translated. (DOCX 13 kb) [file 12872_2015_148_MOESM2_ESM.docx]

Personal ID: _____________-_________

# Register of postoperative infections after cardiothoracic surgery, Uppsala University Hospital.

At the department of Cardiothoracic Surgery in Uppsala there is a continuous follow-up regarding potential wound infections following surgery as a part of our quality improvement work. The wound can even in normal settings secrete a clear fluid which will stop after a while. In cases of infection, the wound secretion continues. The fluid from the wound can also change appearance and become yellowish in color and sometimes start to smell bad. Per definition, pus visible to the eye indicates an infection.

The results from the follow-up provides us with important information that is necessary for us to be able to improve our perioperative routines. By answering the questionnaire You help us to provide the best possible care. Your answers and personal information will be treated confidentially and will not be identifiable in our report. The follow-up adheres to the regulations in the Data Protection Act.

**Questions to those that have been operated in the heart:**

Have you, during or after discharge from your heart surgery, had an infection in the chest wound?

yes no

Have you, during or after discharge from your heart surgery, had an infection in the wound where the drains were placed?

yes no

**The following question is to to be answered by those that have been operated with coronary surgery where a vessel has been taken from the leg:**

Have you, during or after discharge from your heart surgery, had an infection in the wound on the leg?

yes no

**The following question is to be answered by those that have been operated where a vessel has been taken from the arm:**

Have you, during or after discharge from your heart surgery, had an infection in the wound on the arm?

yes no

**The following question is to be answered by those that have been operated in the groin:**

Have you, during or after discharge from your heart surgery, had an infection in the groin?

yes no

**Question to be answered by those operated in the lung:**

Have you, during or after discharge from your lung surgery, had an infection in the chest wound?

yes no

Have you, during or after discharge from your lung surgery, had an infection in the wound where the drains were placed?

yes no

**If you have had an infection and answered yes on any of the above questions we want to know what care You have received.**

Were you readmitted to hospital because of your infection?

yes Which hospital? _____________________ no

Have they taken a bacterial wound culture?

yes no Don’t know

Were you treated with antibiotics?

yes Name of antibiotic: _____________________ no

**Date: _______________ Name: _____________________**
